# Bacterioplankton response to nitrogen and dissolved organic matter produced from salmon mucus

**DOI:** 10.1002/mbo3.1132

**Published:** 2020-11-24

**Authors:** Verónica Molina, Camila Fernández

**Affiliations:** ^1^ Departamento de Biología (Programa de Biodiversidad & Observatorio de Ecología Microbiana) Facultad de Ciencias Naturales y Exactas Universidad de Playa Ancha Valparaíso Chile; ^2^ HUB Ambiental UPLA Universidad de Playa Ancha Valparaíso Chile; ^3^ Centro Interdisciplinario para la Acuicultura sustentable (INCAR) Universidad de Concepción Concepción Chile; ^4^ Centro de Investigación Oceanográfica COPAS Sur‐Austral Universidad de Concepción Chile; ^5^ Laboratoire d'Océanographie Microbienne (LOMIC) Sorbonne Université CNRS Observatoire Océanologique Banyuls/mer France; ^6^ Centro Fondap de Investigación Dinámica de Ecosistemas Marinos de Altas Latitudes (IDEAL) Valdivia Chile

**Keywords:** ammonium regeneration, aquaculture, bacterioplankton diversity, coastal ocean, nutrients, salmon skin mucus

## Abstract

Aquaculture releases organic matter to the water column through excretion, fecal pellets, and uneaten food, but also by the continuous release of fish epithelium mucus. The effect of the latter on natural bacterial assemblages was determined using ammonium amended experiments at Puyuhuapi fjord in Chilean Patagonia. Mucus was added to seawater coming from 2 and 100 m depth and ammonium, nitrite and nitrate, dissolved organic carbon (DOC), picoplankton abundance, and active composition (i‐tag 16S rRNA) were followed for 24 h. The results showed a significant response from the microbial community but only at surface depth after 2 and 6 h of incubation. A reduction of DOC and ammonium concentration and accumulation of nitrite and nitrate over time was observed, mainly at 100 m. Changes in the composition of active bacteria between treatments were observed at different taxonomic levels, associated with Alphaproteobacteria (Clade SAR11), Bacteroidetes (*Polaribacter*) and Gammaproteobacteria (*Colwellia*, *Oceaniserpentilla)* and other bacteria such as *Nitrospina* sp, a nitrite‐oxidizing bacteria at some hours during the incubation. Fish pathogens, such as *Vibrio* and *Piscirickettsia* were rare (<0.02%). Overall, our study suggests that fish mucus can cause rapid modifications in microbial assemblages and stimulate organic matter and nutrient cycling, including heterotrophic and autotrophic (nitrification) in areas influenced by aquaculture.

## Graphical abstract

Salmon farming can modify ambient microbial community structure, including specific groups like marine nitrifying bacteria. Fish mucus represents an additional source of dissolved organic matter and nutrients to the water column. There is a rapid response of fjord free‐living microbial community to mucus addition.

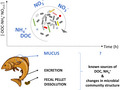

## 1 INTRODUCTION

Mucus is a matrix layer generated by fish epithelium with physical and chemical characteristics that provide the organisms with protection as well as ecological intra‐ and interspecific (even symbiotic) interactions through signaling, among other functions, recently reviewed (Reverter et al., 2018). Early studies described mucus as a primary defense against pathogens, such as parasites, bacteria, and fungi, through a continuous loss and replacement (Fletcher, 1981; Ourth, 1980). However, a plethora of functions have been attributed to the fish mucus, that is, osmoregulator and a barrier to avoid abrasion, toxins, and even UV protector, besides antimicrobial and chemical communication associated with mucins, immunoglobulins, lectins, lysozymes, antimicrobial peptides, among other compounds (Reverter et al., 2018). Moreover, despite antimicrobial activities (Kumari et al., 2019), skin mucus is inhabited by many commensal/opportunistic microbes, reaching higher abundances ~4x10^8^ cell (Bernadsky & Rosenberg, 1992; Minniti et al., 2017). This ecological interspecific interaction within the mucus is generated from early stages in teleost fishes such as Salmon due to microbial colonization from the surrounding ecosystem helping to develop the fish immune system (Kelly & Salinas, 2017). Despite its protective role and potential microbe‐host beneficial interactions, fish mucus is vulnerable to the ability of certain pathogens to adhere and invade (e.g., Zuo et al., 2019).

Recent characterization of the skin mucus microbiome using 16S rRNA sequencing metabarcoding (iTag) demonstrate a predominance of Proteobacteria, Firmicutes, and Acidobacteria, with high intraspecies variability and impact of fish handling on the microbial composition of epithelial mucus (Minniti et al., 2017). The rate at which skin mucus is produced can increase under stress, for example, in the confined conditions related to fish farming (Iger & Abraham, 1997) and parasitic infections (Karlsen et al., 2018). It is expected that under such stress a potentially significant amount of mucus could be released to the surrounding water column.

Salmon farming, as an economically productive system, has been known to generate impacts on local biogeochemical conditions since it constantly adds organic matter and nutrients enrichment to the surrounding water through excretion, fecal pellets, and uneaten food (Navarro et al., 2008). Aquaculture has indeed been related to eutrophication and anoxic biogeochemical conditions particularly in sediments, where an increase in sulfide and ammonium emissions has been reported (Holmer & Kristensen, 1996). These features are associated with disturbances of natural microbial communities by stimulating for example the enrichment of Delta and Epsilonproteobacteria that intervene in sulfur recycling in sediments (Rubio‐Portillo et al., 2019). Also, high abundances of heterotrophic and autotrophic planktonic communities have been reported in areas close to aquaculture centers (Pitta et al., 2006; La Rosa et al., 2002; Sakami et al., 2003) suggesting a potentially active recycling community that could transform mucus into nutrients in short time scales.

Herein, we explore the influence of Salmon mucus enrichments on biogeochemical conditions and the response of natural bacterioplankton communities from Northern Patagonia (Puyuhuapi Fjord, Chile 44° 35,909 ‘S; 72 ° 42,721’ W), an area impacted by the development of salmon farming (Buschmann et al., 2006; González et al., 2011). Using an experimental approach, we evaluated diversity changes and nutrient production fluxes associated with mucus production. Implications for trophic conditions within salmon farming facilities are discussed.

## 2 MATERIALS AND METHODS

### 2.1 Sampling and study area

Two experiments were carried to analyze the potential effect of mucus secretion on nutrient cycling and microbial diversity. During the experiments, sampling was carried out at the Puyuhuapi channel (44° 35,909 ‘S; 72° 42,721 ‘W) and the data was collected during the 2015 cruise to Puyuhuapi Fjord organized by the center COPAS SurAustral. The Sampling site was visited on August 7th of 2016 and water samples were taken from the surface (2 m) and marine waters (100 m). Hydrographic data (temperature, salinity, dissolved oxygen) were obtained using a CTD (Seabird Electronics SBE Model 25).

Ammonium samples were taken in duplicates using 50 mL Duran Schott flasks and analyzed according to the fluorometric method (Holmes et al., 1999). Samples for the determination of nitrate and nitrite were filtered through a 0.7 µm GF/F filter and stored in duplicates in 15 mL falcon tubes (−20ºC) until analyzed by standard colorimetric methods (Aminot & Kérouel, 2007). The concentration of DOC was determined by chromatography using a Shimadzu TOC‐5000 carbon analyzer (Benner & Strom, 1993).

### 2.2 Mucus effect experiments

Extraction of mucus from *Salmo salar* was carried out from live specimens previously anesthetized (3 min exposure) with benzocaine solution 20% P / V (1 mL in 20 L of seawater filtered by 5 µm). Immediately after anesthesia, the fishes were put on a metal tray (previously autoclaved at 120° C for 15 min) and rolled on the metal tray about 15 times per minute to manually stimulate mucus secretion. After extraction of mucus from 18 individuals, the fishes were deposited in a container with seawater without anesthetic for recovery.

The mucus obtained from 18 individuals of *Salmo salar* represented a total volume of 70 mL which was deposited in 50 mL falcon tubes and stored at −80°C. The mucus was thawed at room temperature and then centrifuged at 3200 g and 4°C for 15 min. The supernatant was transferred into a 50 mL falcon tube and centrifugation was repeated to ensure complete removal of the mucus. We obtained a final pellet (1 mL) that was later resuspended in 500 mL of Milli Q water to generate a “mucus solution” to be used as an amendment. Before the experiments, this solution was sterilized by filtration (0.22 μm) to avoid introducing external bacteria onto our natural microbial community.

Microcosms were set up as follows: Eight Nalgene bottles of 10L (previously autoclaved at 120°C; 30 min) were filled with filtered seawater (0.7 μm) coming from 2 and 100 m depth. Two bottles containing 2 m seawater were inoculated with 9.38 mL of mucus solution and two bottles containing water from 100 m depth were amended with 3.1 mL of the same solution. The other sets correspond to controls, without the addition of mucus. The mucus inoculum used for each depth was calculated by estimating the ammonium content of fish mucus as well as its environmental concentrations at both depth levels (2 and 100 m). Mucus additions were then calculated, and amendments were estimated to represent 10% of ambient concentrations and avoid excessive enrichment. Incubations were performed at room temperature (14° C) under artificial light and lasted for 24 h. Intermediate sampling times were done at 2, 6, and 12 h. A volume of 500 mL was taken through dispensers attached to the Nalgene bottles for nutrients and dissolved organic carbon. Also, samples were collected to determine bacterial abundance and filtration for molecular analyses for each sampling time. Bacterial abundance was determined by flow cytometry. For doing so, 1 mL of seawater was fixed using glutaraldehyde (final concentration, 0.1% v/v) in duplicate cryovials, stored in liquid nitrogen in the field, and then at −80 ºC, until analyses (FACSCaliburTM flow cytometer, Becton Dickinson Biosciences, CA, USA).

A Spearman Correlation Rank was carried out with cell abundance, DOC, and nutrient analyses. Two‐way (ANOVA) analysis was carried out to evaluate the significance of the differences between treatments during the time course of the experiment. Shapiro‐Wilk test was used to determine normality distribution. Then a pairwise multiple comparisons and a posteriori Tukey test were estimated using GraphPad Prism. Significance was considered when *p* < 0.05.

### 2.3 Molecular and bacterial community analyses

Bacterioplankton was concentrated by filtration (100 mL seawater) onto hydrophilic PVDF filters (0.22 μm; GVWP02500, Millipore, 25 mm diameter) then the filters were preserved with RNA‐later (300 μL) and stored frozen (−20ºC) until extraction. RNA was extracted with the mirVana miRNA Isolation Kit (Ambion, Life Technologies) following the specified protocol but with the addition of a mechanical disruption step during the cell lysis phase as described previously (Valdés et al., 2017). RNA extracts were then treated to remove DNA tracers using TURBO DNA‐free Kit (Ambion) and quantified using a fluorometric method using Qbit (Thermo Fisher Scientific). Complementary DNA (cDNA) was generated using ImProm II™ Reverse Transcription System (Promega Corp, Madison, WI) and amplification was checked using standard PCR to amplify V1‐V3 region of bacterial 16S rRNA gene 28F (5′‐GAG TTT GAT CNT GGC TCA G‐3′) and 519R (5′‐GTN TTA CNG CGG CKG CTG‐3′). Using the same primers, cDNA templates were sequenced with MiSeq (Illumina) at the Molecular Research LP (Mr. DNA, www.mrdnalab.com, Texas, USA).

Sequence reads were filtered for primers and barcodes, then short and low‐quality sequences (<200 bp, ambiguities, and maxima of 8 homopolymers) were removed using MOTHUR software v1.35.1 (Schloss et al., 2009). The bacteria taxonomic affiliations were determined using SILVAngs (v128) database pipeline available from https://www.arb‐silva.de/ (Quast, Pruesse, and Yilmaz 2013). The libraries were deposited in the European Nucleotide Archive under the study accession number PRJEB32384 (40 paired reads ERS3391671 ‐ ERS3391710).

Alpha and Beta diversity analyses of the bacterioplankton community structure were analyzed using the software PRIMER (7.0.11) with the PERMANOVA add on (Anderson et al., 2008). The Alpha diversity, including nonparametric indices such as richness (S), Shannon (H’), and Evenness (J) were estimated, and Rarefaction analyses were included to compare the different libraries considering sequencing depth normalization to the library having the lower number of sequences (see Appendix file). Principal Coordination Analyzes (PCoA) were carried out to determine changes in the microbial community structure associated with mucus enrichment at both depth levels. This analysis was performed after transforming the data and based on a similarity matrix (Bray‐Curtis). To visualize changes associated with specific microbial communities Pearson correlation analyses were overlaid PCoA, vectors representing significant correlations (R > 0.7). To determine the taxa (phyla and OTU level) with a higher contribution associated with the different treatments, a SIMPER analysis was performed using PRIMER.

## 3 RESULTS

**FIGURE 1:**
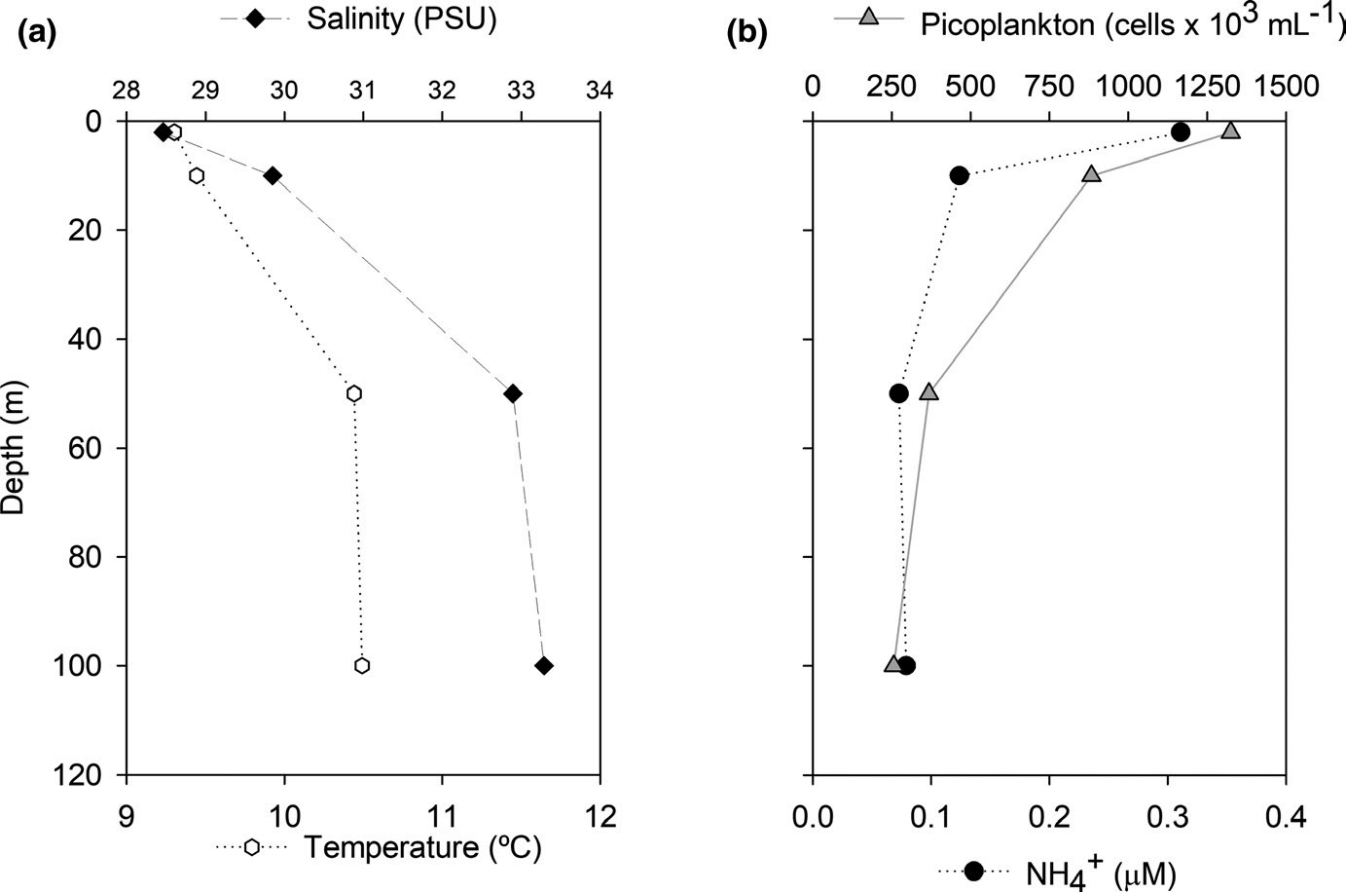
Hydrographic variables at Puyuhuapi Fjord. Profiles of temperature, salinity, ammonium concentrations, and bacterial abundances between 2 and 100 m depth.

Hydrographic conditions during sampling are shown in Figure 1a. Temperature and salinity increased from values close to 9°C and 25.8 psu in surface waters to 10.5ºC and 33.3 psu at 100 m, respectively, which is consistent with the estuarine conditions at the fjord (estuarine waters in the surface layer and marine waters at depth). Ammonium concentrations, on the other hand, decreased with depth from 0,311 μmol L^−1^ at 2 m to 0.051 μmol L^−1^ at 50 m. Concentrations reached 0.079 μmol L‐1 at 100 m depth (Figure 1b). Coincidently with ammonium, picoplankton abundance (Figure 1b) was characterized by higher values in surface waters (1,324 x 10^3^ cell mL^−1^ at 2 m depth) compared with cell abundances observed at 100 m (257 x10^3^ cell mL^−1^).

### 3.1 Biogeochemical changes response to mucus amendments during incubations

**FIGURE 2:**
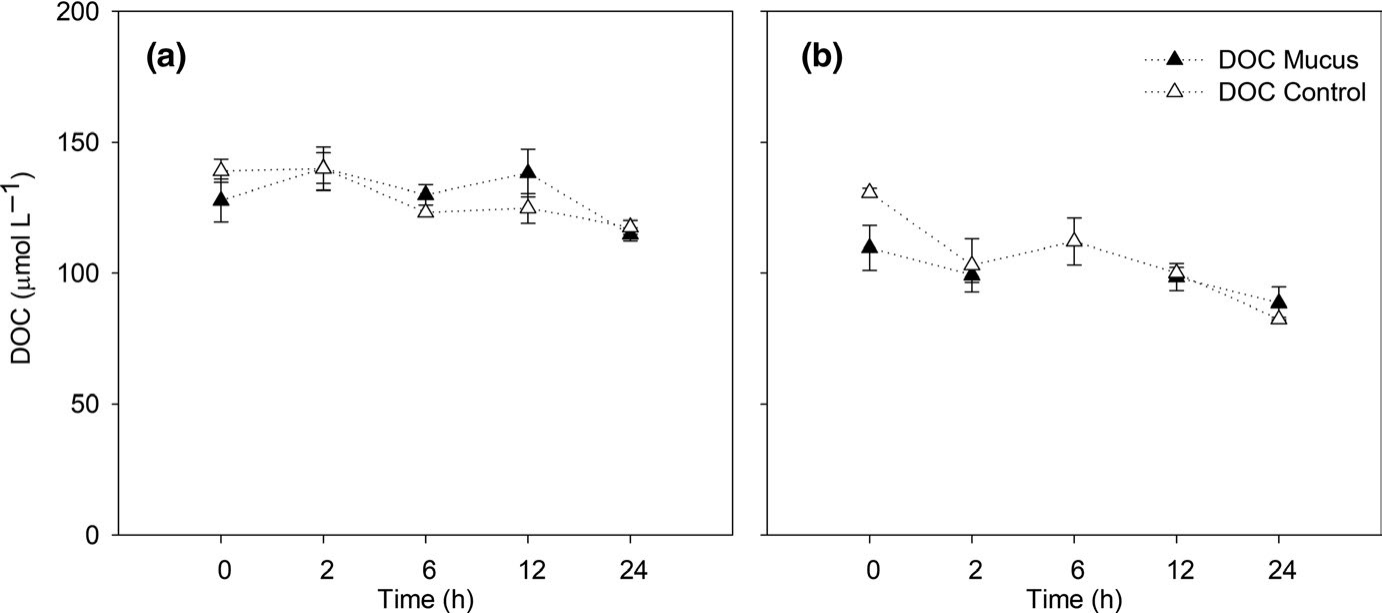
Temporal evolution of dissolved organic carbon (DOC). Trends are described for experiments at a) 2 m depth and b) 100 m depth.

DOC concentration changes during the experiments at 2 m depth are shown in Figure 2a. In the mucus‐amended experiments, a 19.2% increase between T0 and T2 ‐T6 was observed followed by a ~ 17% decrease between T12 and T24. In contrast, the control showed a slight decrease from T0 reaching the lowest values, as the amended experiment at T24. In contrast, DOC concentration changes through time at 100 m depth were similar between mucus amended and the control, except at T0 (Figure 2b). In general, DOC concentrations decreased by 21% between the T0 and T2. The values between T6 and T24 showed a decrease of 17.5% for both treatments.

**FIGURE 3:**
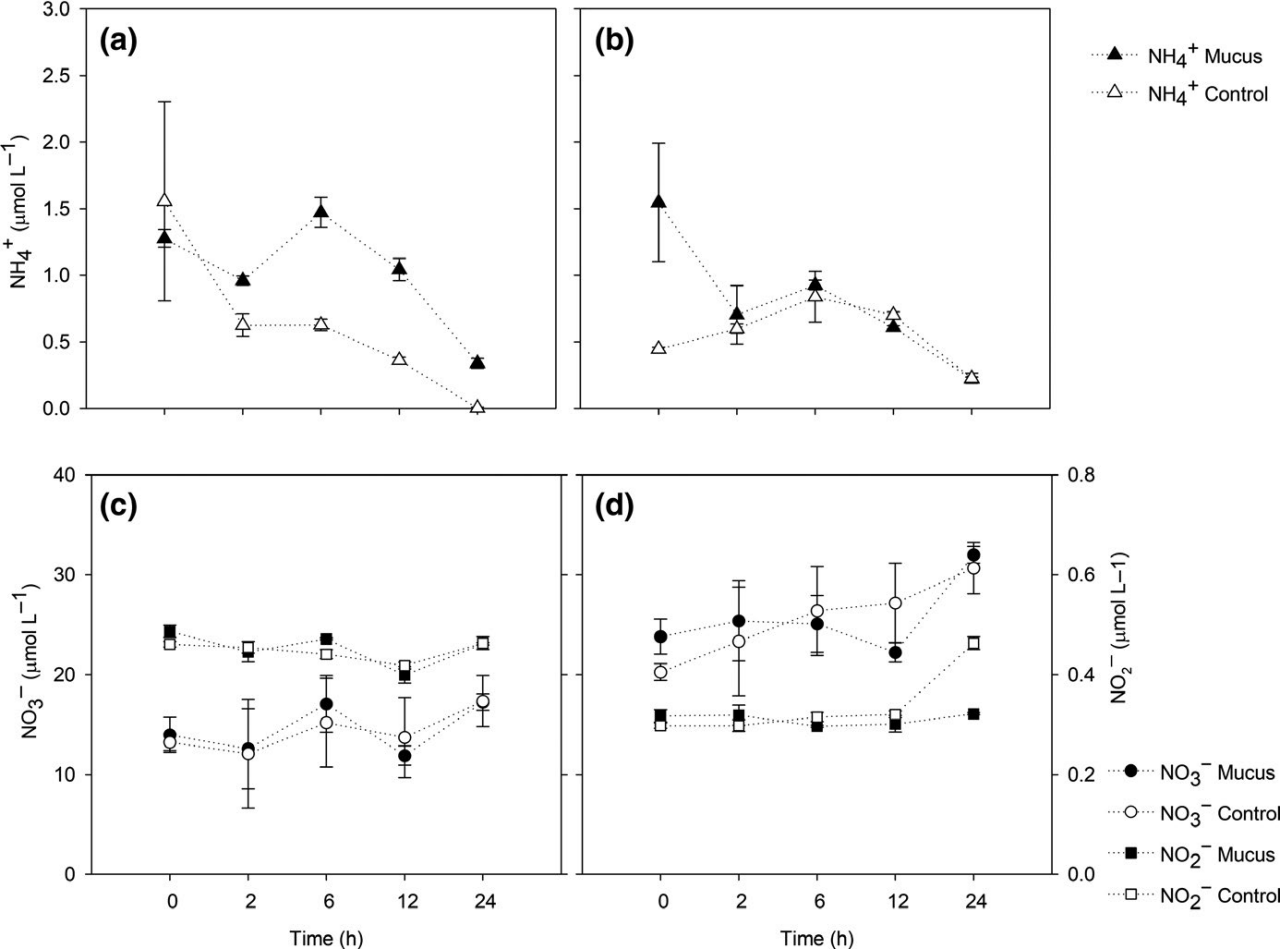
Temporal evolution of nutrients. Concentrations for mucus and control treatments. a) Ammonium at 2 m, b) Ammonium at 100 m, c) Nitrate and nitrite at 2 m and d) Nitrate and nitrite at 100 m.

Ammonium concentrations in the experiment at 2 m showed higher values in mucus‐amended treatment compared to the control (Figure 3a) except at T0. From T0 to T2, a decrease of 24% was observed in the mucus‐amended treatment followed by an increase of 53.4% between T2 and T6. Between T6 and T24, ammonium values decreased through time up to 0.34 ± 0.25 μmol L^−1^ (Figure 3a). In the control treatment, ammonium concentrations decreased by 60% between T0 and T2‐T3 and then decreased through time reaching 0 at T24. For the experiment at 100 m, ammonium values remained similar between the treatment and control until T24 with the exception to T0 (Figure 3b), where mucus addition generated a slight ammonium enrichment. In the mucus‐amended treatment, a decrease of 54% was observed between T0 and T2. Then a 31.7% increment was observed between T2 and T6 followed by a decrease of 76% until T24. For the control treatment, a sustained increase of 88% between T0 and T6 was observed.

Nitrate and nitrite concentration changes in the 2 m experiments with mucus enrichment were similar to the control (Figure 3c). Nitrate and nitrite change through time was characterized by a decrease from T0 to T2, then an increase from T2 to T6, and again consumption occurred between T12 ‐ T24. A slightly higher accumulation of both nutrients was observed at T6 in mucus‐amended treatments. In contrast to the 2 m experiment, the 100 m incubation (Figure 3d), showed a higher nitrate accumulation over time, with a clear trend in the control compared to the amended treatment. Nitrate concentration reached the highest concentration (~30 μmol L^−1^) at T24 in both treatments. The concentration of nitrite remained similar between the two treatments over time (Figure 3d) except for T24 for the control in which a 0.2 μmol L^−1^ increase was observed. For the treatment with mucus, concentrations remained close to 0.5 μmol L^−1^ with no significant variation over time.

The two‐way ANOVA analysis carried out for 2 and 100 m depth showed that no significant variations were found between DOC, ammonium, and nitrate a concentration changes between treatments and incubation time (*p* > 0.05). Differences between treatments were observed at *p* = 0.06 only for DOC, whereas DOC and nitrate presented significant differences between the incubation hours (DOC, *p* = 0.01, between T0 and T24, and nitrate, *p* < 0.04 (between T0, T2 and T12 compared with T24).

### 3.2 Active microbial community structure and composition response to mucus addition

**FIGURE 4:**
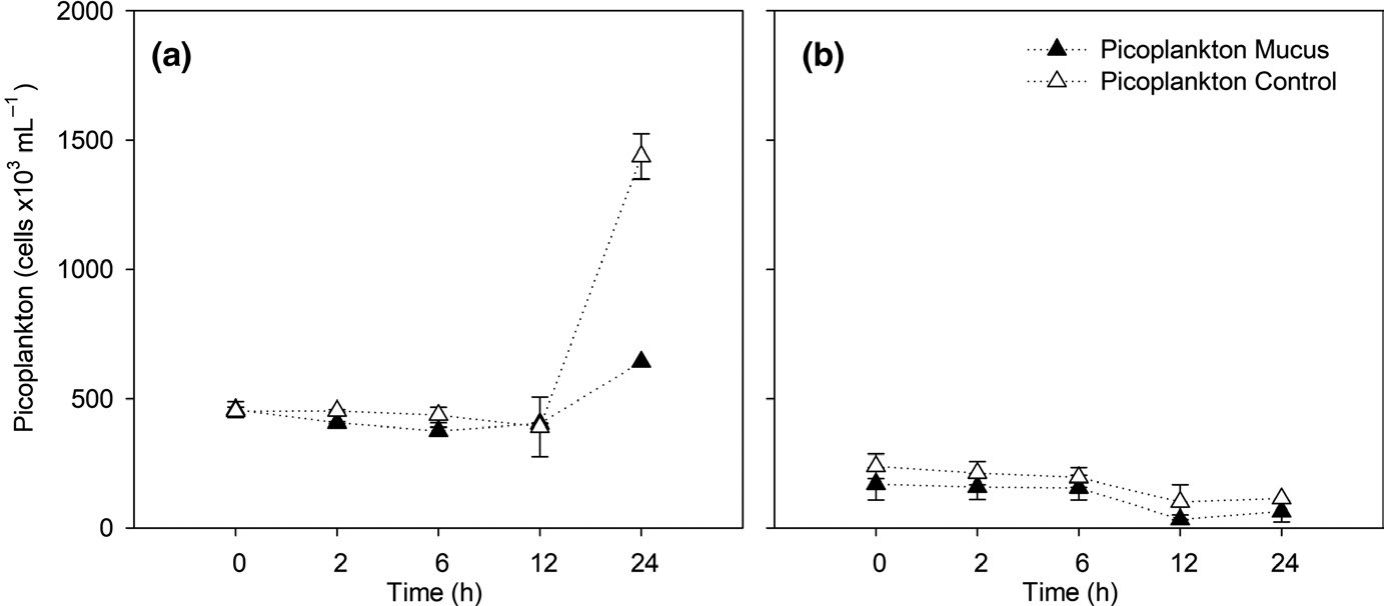
Temporal evolution of cell abundance during the experiments at a) 2 m and b) 100 m depth.

Picoplankton cell abundances changes determined during the experiments are shown in Figure 4. The picoplankton abundance was higher at 2 m compared with the 100 m depth experiment. At 2 m and 100 m experiments the abundances were almost invariant through time, except for a bloom that was observed between T12 and T24 only at 2 m depth (Figure 4a), characterized by >50% cell increase at the control and mucus‐amendment treatments reaching up to 1437.56 ± 87.6 x 10^3^ and 643.21 x 10^3^ cell mL^−1^, respectively. The two‐way ANOVA presented no significant variability between treatments at each depth through time (*p* > 0.05). The cell abundances were significantly (*p* < 0.001) correlated with DOC, nitrate, and nitrite (R = 0.75, −0.76, 0.62, respectively).

**FIGURE 5:**
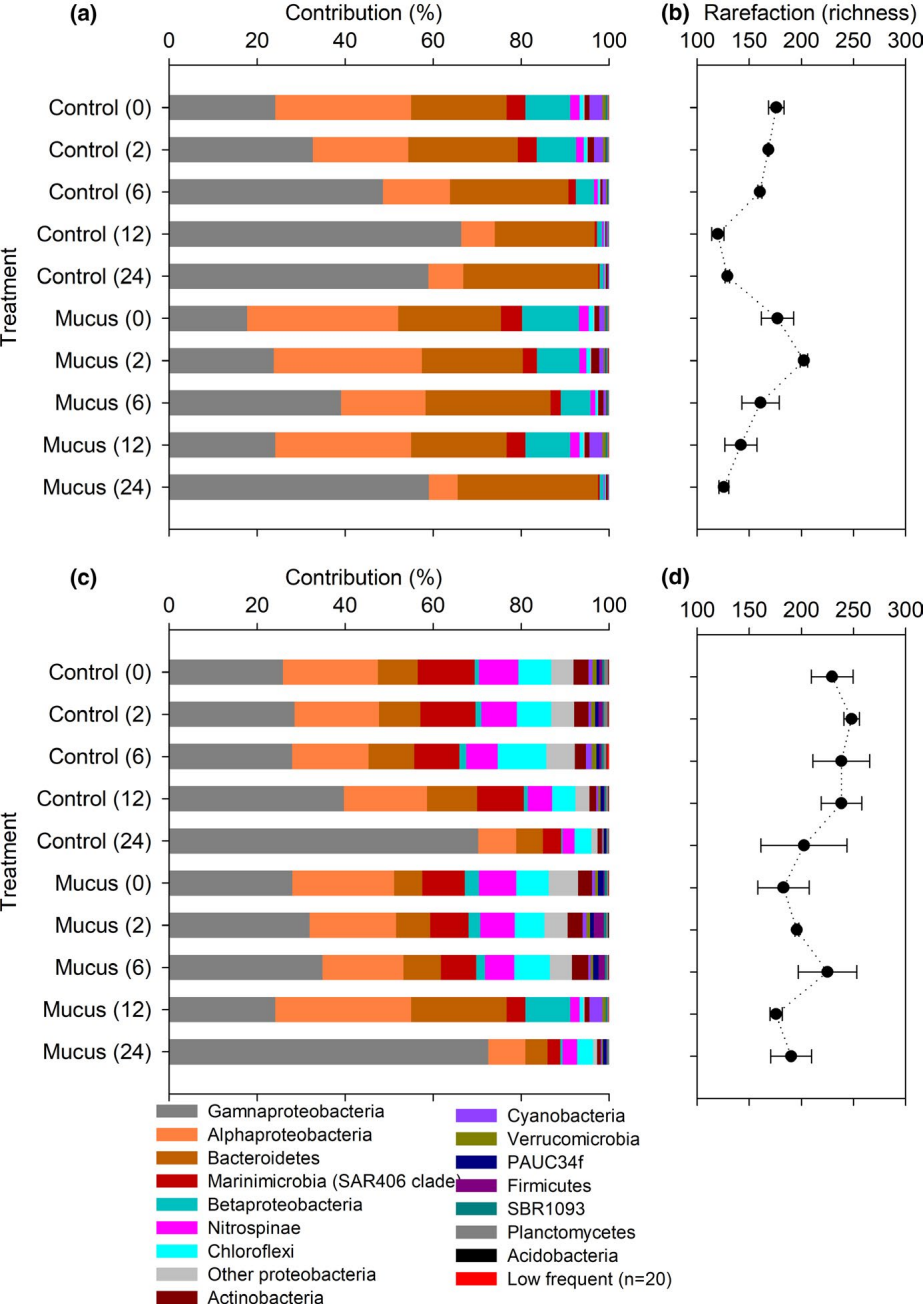
Contribution and normalized richness of bacterioplankton over time (at taxa level) during the experiments at 2 m (a and b) and 100 m depth (c and d).

The contribution (percentage) of predominant phyla and Proteobacteria classes based on the percentage estimated from the sum of both replicates 16S rRNA libraries per incubation time (see Appendix file) are shown in Figure 5. The initial composition of active bacteria in both experiments (2 and 100 m depth) was characterized by the predominance of Proteobacteria (Gamma‐ and Alphaproteobacteria classes), followed by Bacteroidetes at 2 m depth and Marinimicrobia (SAR406 clade) at 100 m depth (Figure 5a and 5c). Less predominant was Betaproteobacteria at 2 m depth (Figure 5a) and Nitrospinae, Chloroflexi, and Planctomycetes phyla at 100 m depth (Figure 5c). In general, the 100 m depth sample showed a richer bacterial community composition compared to the 2 m depth (Figure 5b and 5d).

Changes in the active bacterial composition were observed in both experiments through time, but they were notorious mostly at 2 m incubation (Figure 5a). In the 2 m depth control treatment, Gammaproteobacteria and Bacterioidetes were more abundant toward the end of the incubation (>15%, Figure 5a) whereas the rest of the bacterial community showed decreasing richness over time (Figure 5b). In contrast, the 2 m depth mucus‐amended treatment showed a higher evenness in the bacterial composition with a slightly higher contribution of Alphaproteobacteria and Betaproteobacteria compared to the control (Figure 5a). This variability was associated with an increase in richness during the first two hours (T0 ‐ T2) of incubation. However, both control and mucus‐amended treatments were similar in their active bacteria contribution toward the end of the experiment (T24).

In the 100 m depth experiment, bacteria community composition was comparable between the control and mucus‐amended treatment through the incubation during the first six hours (Figure 5c). However, at T12 and T24 in the 100 m depth control experiment, Gammaproteobacteria presented an increasing contribution in the 16S rRNA libraries. Whereas in the mucus‐amended experiment a higher contribution of Bacteroidetes was observed at T12 and of Gammaproteobacteria only at T24 (Figure 5c). These later changes in bacteria composition were also observed in richness decrease toward the end of the incubation (Figure 5d).

**FIGURE 6:**
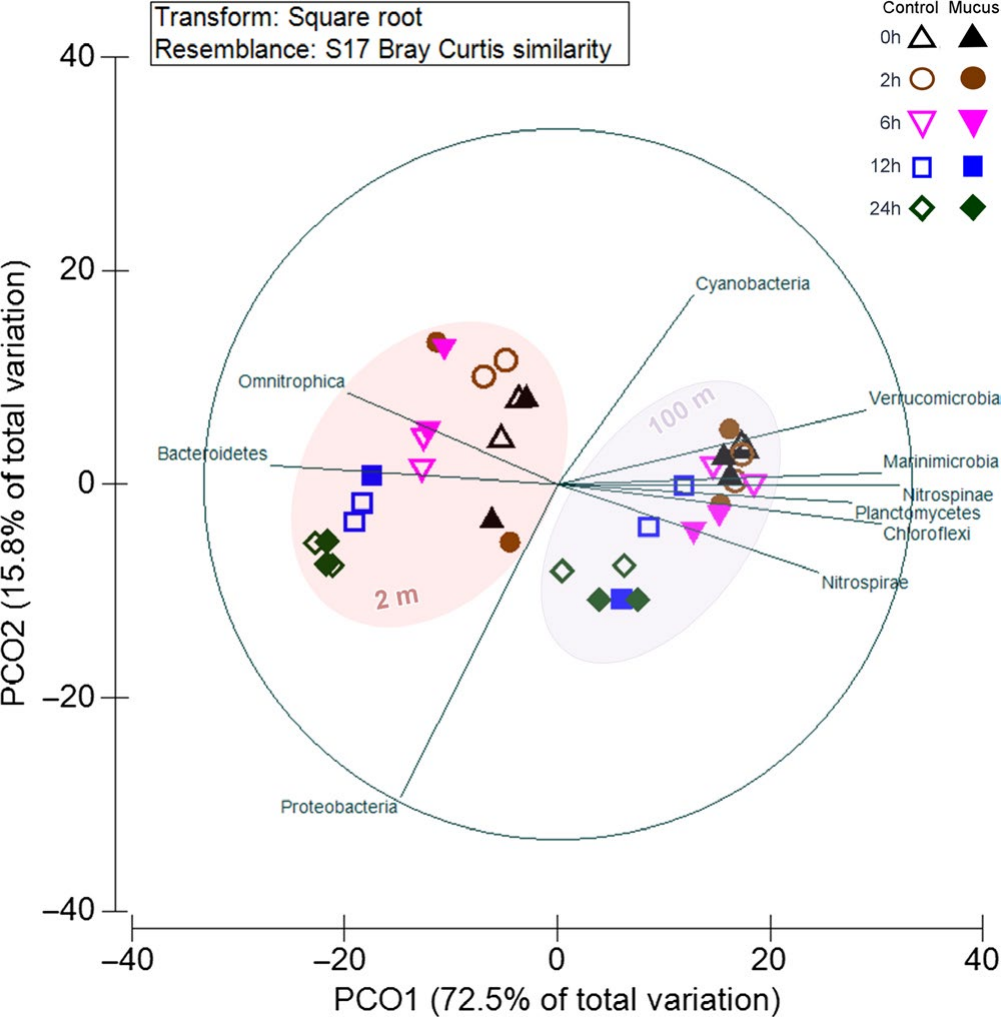
Principal Coordinated Analyses (PCoA) showing bacterial community variability associated with the different treatments (control versus mucus) through time in both depths. Phyla having significant correlations (R > 0.7) were also plotted.

Principal Coordinate Analysis, (PCoA, Figure 6) estimated using all 16S rRNA libraries replicates showed a great similarity between all the active bacteria community structure of the 100 m depth experiment, but with variability associated with the incubation time T24. PCoA evidenced a higher variability in the active bacterial community structure from the 2 m depth experiment, including the separation of the first incubation times (T0 ‐ T6) from the last (T12 and T24) and mucus amendment, particularly for T2. Most of this variability was associated with the order Alteromonadales from Gammaproteobacteria class, Bacteroidetes associated with Flavobacteriales order, Marinimicrobia, and Nitrospinae, based on a SIMPER analysis (showing between 7 and 23.8% dissimilarity between both amended treatment versus control). Both, Bacteroidetes and Proteobacteria phyla also presented significant differences based on a two‐way ANOVA result and Tukey`s multiple comparison test (*p* < 0.05) mostly associated with time within each experiment and between treatment and control at 2 and 6 h of incubation associated with Bacteroidetes and Proteobacteria for both depths and Chloroflexi, Marinimicrobia and Nitrospinae at 100 m depth.

**FIGURE 7:**
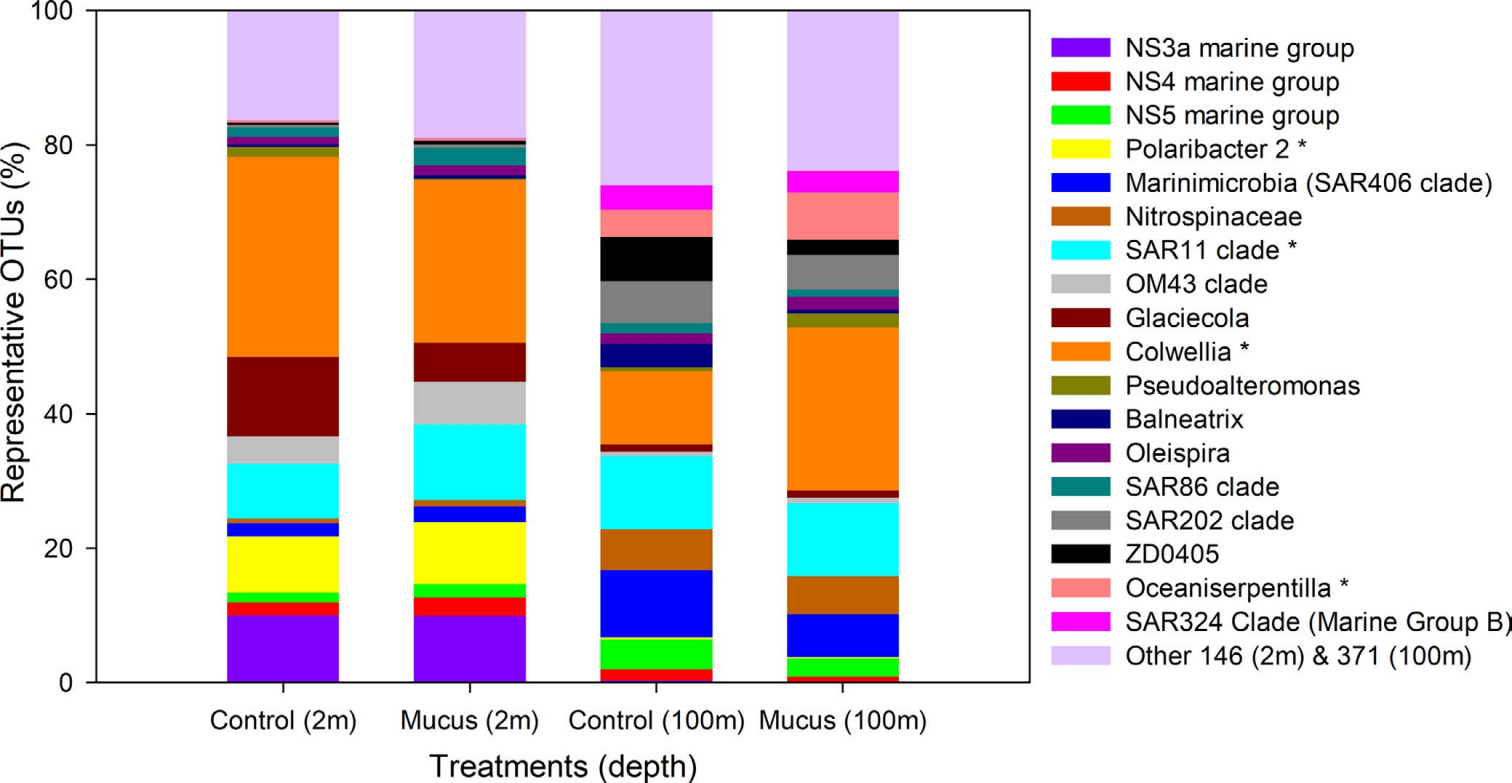
Comparison of OTUs showing >4% contribution based on SIMPER analyses in the different treatments and depths.

**FIGURE 8:**
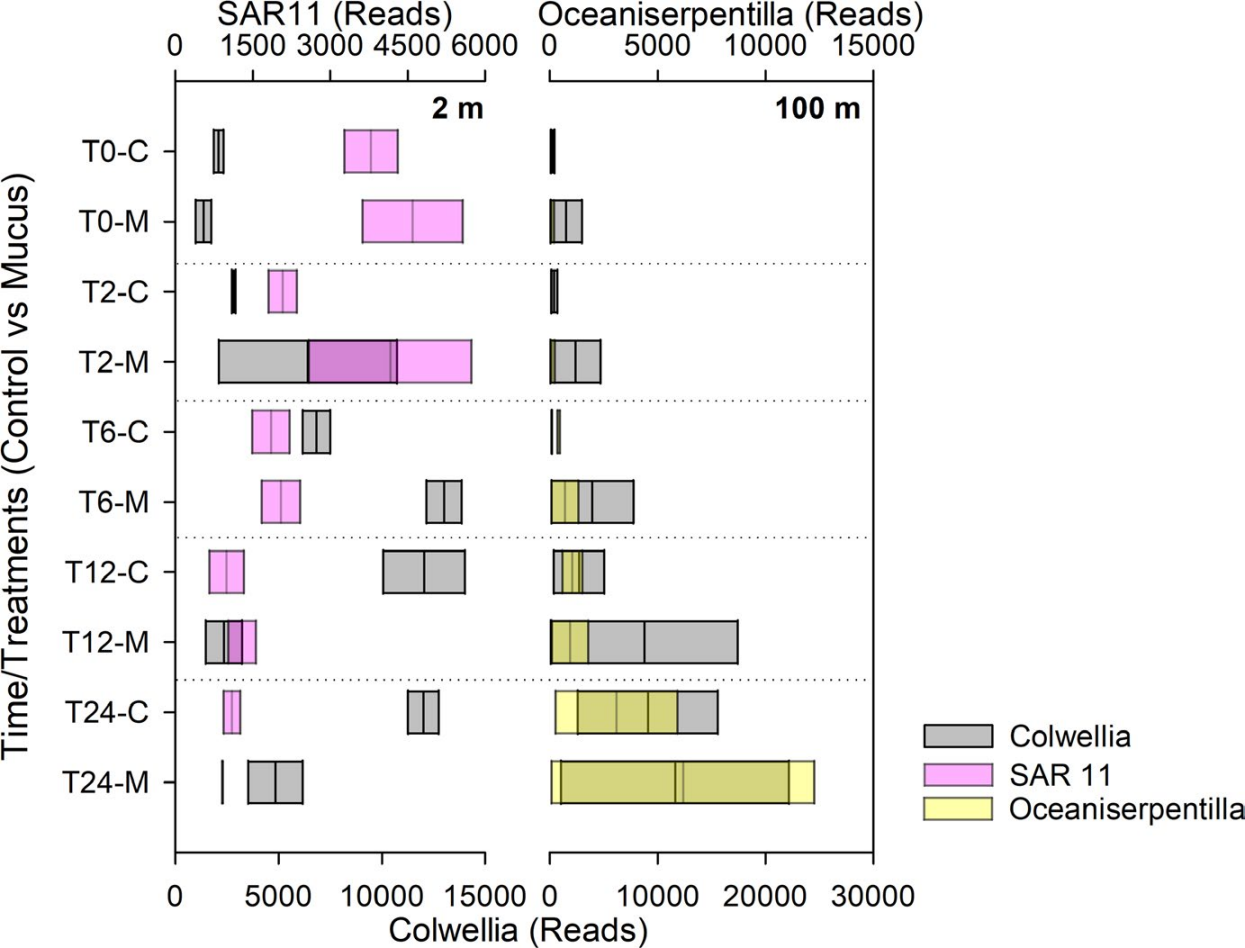
Boxplot of selected OTUs at different depths showing >4% contribution based on SIMPER analyses dissimilarity between control and mucus treatment variability through time (considering replicates).

**FIGURE 9:**
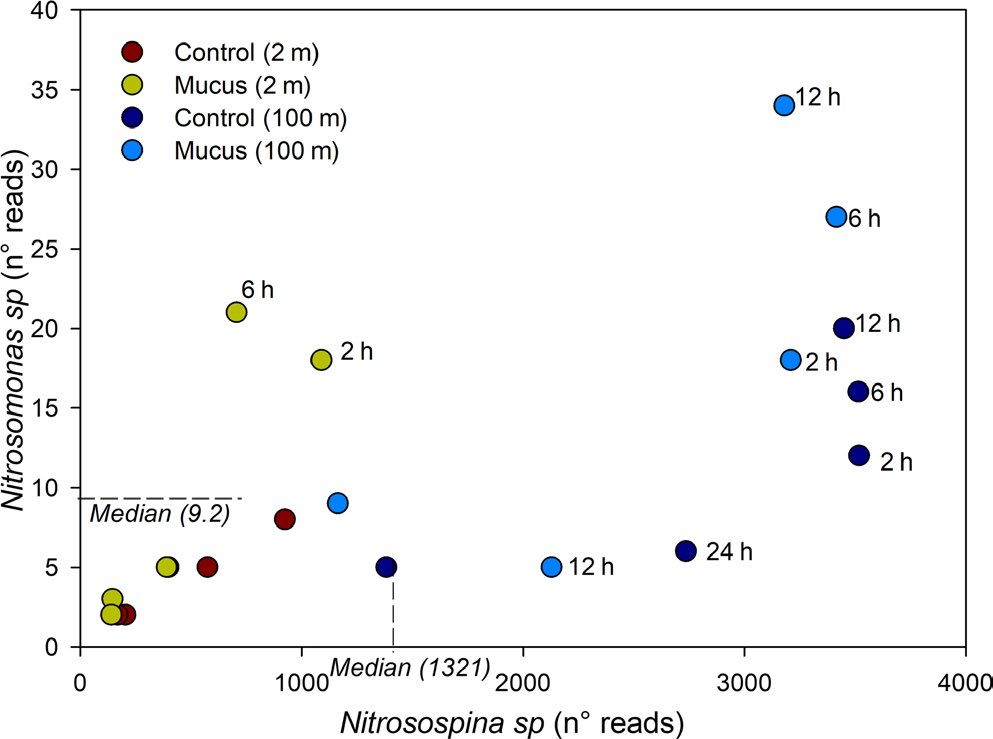
Abundance and activity of functional groups associated with ammonium oxidation during mucus addition experiments.

**FIGURE 10:**
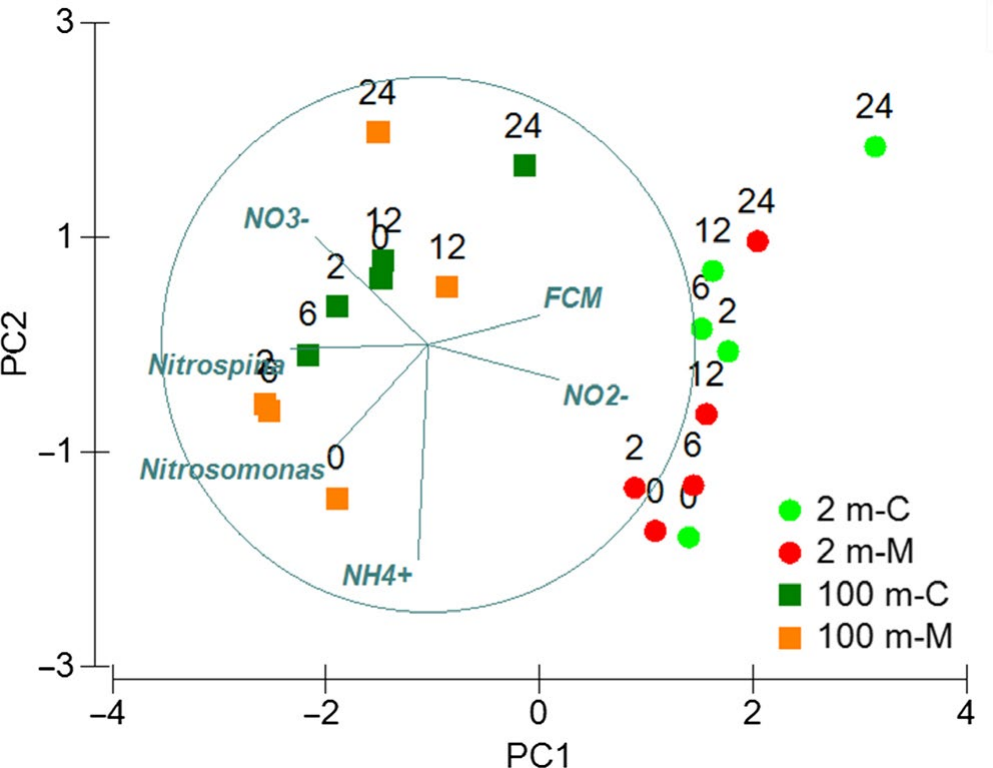
Principal Component Analyses (PCA) associated with different chemical variables, abundances (flow cytometry), and reads related to nitrifying microbial communities.

At a low taxonomy level, SIMPER analysis indicates that 18 OTU presented >4% the contribution of the different experiments and depths, individually represented >0.2% of contribution to the total sequences reads grouped by treatment (Figure 8). This analysis indicates that OTUs contributing >4% to the dissimilarity between treatments were affiliated to *Colwellia*, SAR11 clade, *Polaribacter* at 2 m depth, and *Colwellia*, *Oceaniserpentilla*, and *Belneatrix* at 100 m. These analyses also discriminate against other OTUs relevant in one depth versus the other including fish mucus and nitrogen recycling microbes, that is, *Nitrospina sp*, a nitrite‐oxidizing bacteria (Figures 7 and 8). The activity of nitrifying bacteria reached higher frequency in 16S rRNA libraries for 100 m depth compared with 2 m depth experiments. Moreover, the comparison of *Nitrospina sp* versus *Nitrosomonas sp* indicates that higher numbers both presented a greater enrichment in mucus‐amended experiments, suggesting a role in nitrate accumulation predominantly in subsurface waters (Figures 7 and 9). Known fish pathogens were detected in low numbers, for example, *Vibrio* (<0.02%), *Tenacibaculum* (<0.1%), OTUs associated with *Piscirickettsiaceae* (<0.001%). Also, a Principal Component Analysis of nutrient variability, picoplankton abundance, and known functional groups associated with ammonia and nitrite oxidation (*Nitrosomonas* and *Nitrospina*, ammonia, and nitrite oxidizers) are shown in Figure 10. This analysis indicates that most of the PCA variation (77%) was associated with changes through time in the concentration of ammonia (R = 0.82 with PC2) and nitrate (R = 0.5 with PC1).

## 4 DISCUSSION

This study was focused on identifying the potential for mucus‐driven nutrient enrichment in the environment and its effect on natural microbial communities from aquaculture influenced fjord in Northern Patagonia (Buschmann et al., 2006). The conditions in seawater were associated with the estuarine circulation with low salinity waters at the surface (2 m) and marine conditions at deeper water (100 m) as has been previously described in the Puyuhuapi Fjord (Pérez‐Santos et al. 2017). The addition of mucus produced by Salmon epithelium triggered changes in nutrient dynamics in the estuarine surface as well as marine waters in a deeper layer. These changes were associated with stimulation of the mineralization activity. Ammonium and nitrate changes were consequently observed through time in parallel with significant composition shifts studied through iTag 16S rRNA.

The mucus amendment was designed to provide less than 10% of available ambient ammonium to avoid an overestimation of remineralization rates, allowing a semi‐realistic observation of the changes produced by the presence of mucus. This amendment resulted in a short‐lived variability in ammonium and DOC concentrations (~2 −6 h), resulting in higher concentrations of ammonium in the treatment amended with mucus (1.47 μmol L^−1^, Figure 4) compared to the control. Then, ammonium concentrations decreased toward the end of the incubation. This trend and shifts were attributed to an increase in mineralization of reduced nitrogen (proteins and amino acids) and polysaccharides (glucose) associated with the mucus matrix enrichment. The microbial community response was more evident at 2 m depth where an increase in picoplankton abundance was observed at T24. Many studies have shown the response of bacterioplankton to labile organic matter addition including glucose and the availability of free amino acids as primary drivers of bacterial growth and consequent biomass increase. Therefore, during our experiments, a stimulation of microbial activity was produced, mainly via DOC shifts that resulted in an increased nitrogen demand. Indeed, the observed ammonium produced in the treatment with mucus (Figure 3) was rapidly consumed and after 24 hours, levels decreased to values close to those observed in the control.

Ammonium can be used by microorganisms with different metabolisms including chemoautotrophs and heterotrophs (Falkowski, 1997). Bacterial communities’ growth has been reported in coastal (Pinhassi et al., 1999) and oceanic oligotrophic sites (Liu et al., 2014), independently of the source and complexity of the organic matter available (algal exudates versus glucose, for instance) at a daily scale. On the other hand, our experiments showed evidence of mineralization associated with nitrate and nitrite accumulation in the experiments (Figure 3). This result indicates that ammonia and nitrite oxidation (nitrification) were active during the incubations in both control and mucus‐amended treatment, with higher accumulation at 100 m compared with 2 m depth, earlier in the incubation associated with mucus treatment. Nitrifying microbial assemblages are known to rapidly respond to changes in substrate availability, and it is expected to be down‐regulated by phytoplankton substrate competition mainly at 2 m depth as it is the case in other sunlit areas of the ocean (Smith et al., 2014). Nitrifying communities at fjord ecosystems have been reported to increase their potential activities after ammonium availability was enhanced in the vicinity of salmon farms (Elizondo‐Patrone et al., 2015) and response to food pellet dissolution (Fernández et al. 2020).

Bacterioplankton composition analyses evidenced changes during our experiments associated with time and mucus amendment only during a short scale of incubation (Figure 6). The bacterial community structure based on rarefying richness evidenced a decrease with time for the control and mucus‐amended treatment. Proteobacteria and Bacteroidetes phyla presented a greater contribution to our libraries (16S iTag) and were the phyla showing significant differences in both experiments. Also, the addition of mucus increased the percentage of Alphaproteobacteria. Gamma‐, Alphaproteobacteria, and Bacteroidetes are known for their ability to take better advantage of dissolved organic matter (DOM) referred to as opportunistic heterotrophic marine microbes usually following algal blooms in the ocean (Teeling et al., 2012) and in experimental DOM enrichment experiments, that is, copepod excretion under different trophic conditions (Valdés et al., 2018) or linked to jellyfish blooms (Hao, Wichels, and Fuchs 2019). Also, Bacteroidetes, including the Flavobacteriales order are decomposers of chitin and large polysaccharide, also having the ability to generate biofilms and some are well‐known fish pathogens (Duchaud, Boussaha, and Loux 2007). Mucus‐amendment treatments shaped changes in the microbial community, showing slightly higher richness during the first 6 h of incubation and more notorious at 2 m depth (Figure 6). OTUs characterized by slightly higher contribution in mucus treatment, for example, *Colwellia*, *Polaribacter*, *Pseudoalteromonas*, *Oceaniserpentilla*, at some hours and depths (Figures 7, 8) were previously reported in experiments related with mucus associated with fish (Minniti et al., 2019) and corals (Badhai et al., 2016). In contrast, to the previously mentioned reports, in our study, known fish pathogens such as *Vibrio* were in low numbers (<0.1%). *Colwellia* was a predominant genus associated with differences between treatments found in both depths, responding to mucus addition positively at 6 h and 12 – 24 h of incubation at 2 and 100 m, respectively, a microbe associated with cold marine environments also heterotrophic (e.g., Techtmann et al., 2016).

Considering other OTUs, the specific increase of Betaproteobacteria (*Nitrosomonas*) and *Nitrospina*, both nitrifying bacteria, the first an ammonia oxidizer, and the second a nitrite oxidizer, were indicative of a potentially functional influence effect of mucus enrichment to nutrient recycling (Figures 7 and 9). A higher contribution of both functional groups in the 16S iTag libraries was observed at mucus‐amended treatment compared with the control, mainly at 100 m depth. This result agrees with the observed accumulation of nitrate during the experiment and the variability of *Nitrosomonas* and *Nitrospina* that responded to nutrients based in PCA visualization mainly at 100 m depth (Figure 10).

Salmon mucus in the environment represents a source of organic matter, which in farming centers get released at variable rates because of different factors, such as parasitic infections (e.g., *Caligus rogercresseyi*,) as well as changes in nutrition (Olsen et al., 2008). Moreover, excessive densities within cages could enhance mucus release, thus enriching the water column surrounding areas, stimulating mineralization, and bacterial growth. Moreover, mucus could induce changes in the local microbiome, enhancing heterotrophic groups, and potential fish pathogens.

## 5 CONCLUSION

This study showed that fish mucus represents a source of inorganic nitrogen (ammonium, nitrate, nitrite) and dissolved organic carbon to the water column, stimulating rapid mineralization that includes potential nitrification. Mucus also induced changes in bacterioplankton community structure and composition, with main effects associated with known marine heterotrophic bloomers (Gamma‐ and Alphaproteobacteria and Bacteroidetes, related to *Colwellia*, microbial communities and with functional groups related to marine nitrifying bacteria mainly in subsurface waters.

## ETHICS STATEMENT

This study was approved by the Ethics committee of the University of Concepcion, as part of the FONDECYT Project No. 1180954.

## CONFLICT OF INTEREST

None declared.

## AUTHOR CONTRIBUTION

**Veronica Molina:** Conceptualization (equal); Data curation (supporting); Formal analysis (equal); Investigation (supporting); Methodology (equal); Resources (supporting); Validation (equal); Writing‐original draft (equal). **Camila Fernandez:** Conceptualization (lead); Formal analysis (equal); Funding acquisition (lead); Methodology (lead); Project administration (lead); Resources (equal); Writing‐original draft (equal).

## Data Availability

The sequence data that support the findings of this study are available in the NCBI repository under accession number PRJEB32384 (40 paired reads ERS3391671 ‐ ERS3391710): https://www.ncbi.nlm.nih.gov/bioproject/PRJEB32384. Supplementary table with alpha diversity analyses has been deposited in Zenodo: https://doi.org/10.5281/zenodo.3976815
